# Deep FS: A Deep Learning Approach for Surface Solar Radiation

**DOI:** 10.3390/s24248059

**Published:** 2024-12-18

**Authors:** Fatih Kihtir, Kasim Oztoprak

**Affiliations:** Department of Computer Engineering, Konya Food and Agriculture University, Konya 42080, Turkey; fkihtir@gmail.com

**Keywords:** forecasting, deep learning, feature selection, solar surface exposure, CNN

## Abstract

Contemporary environmental challenges are increasingly significant. The primary cause is the drastic changes in climates. The prediction of solar radiation is a crucial aspect of solar energy applications and meteorological forecasting. The amount of solar radiation reaching Earth’s surface (Global Horizontal Irradiance, GHI) varies with atmospheric conditions, geographical location, and temporal factors. This paper presents a novel methodology for estimating surface sun exposure using advanced deep learning techniques. The proposed method is tested and validated using the data obtained from NASA’s Goddard Earth Sciences Data and Information Services Centre (GES DISC) named the SORCE (Solar Radiation and Climate Experiment) dataset. For analyzing and predicting accurate data, features are extracted using a deep learning method, Deep-FS. The method extracted and provided the selected features that are most appropriate for predicting the surface exposure. Time series analysis was conducted using Convolutional Neural Networks (CNNs), with results demonstrating superior performance compared to traditional methodologies across standard performance metrics. The proposed Deep-FS model is validated and compared with the traditional approaches and models through the standard performance metrics. The experimental results concluded that the proposed model outperforms the traditional models.

## 1. Introduction

Renewable energy represents an environmentally sustainable power source that minimizes CO_2_ emission. Solar and wind power represent primary renewable energy sources with virtually unlimited potential [1]. Today, climate change has become a serious concern, and renewable energy can act as a solution to this. Because they do not emit gases, they reduce carbon footprints and reduce environmental impacts. The depletion of fossil fuels has also contributed to this. Among the available renewable energy sources, solar energy for the generation of electricity [2] has become very popular. As the rapid growth of these cells is increasing, the efficiency has improved greatly, making it more economical and sustainable. With rapid growth and cost efficiency expected, it is likely to become the main energy source in the world.

Solar energy has numerous contemporary applications. There are various environmental parameters [3] that hinder traditional approaches to predicting the solar surface accurately. Deep learning models have larger advancements over the traditional approaches in predicting the time-series data and overcoming environmental parameters such as cloudy skies and orbital positions. By integrating deep learning models, the accuracy of prediction has increased significantly.

Solar power generation depends on weather conditions. This feature destabilizes the overall operation of power systems. This instability can increase power outages and prices, which will adversely affect the cost. Therefore, a technology that can provide a balance between power supply and demand and establish a stable connection between them is required. The conversion of solar energy is performed directly and is related to solar surface exposure. Consequently, variables that are directly related to it have been used to predict the amount of solar power generation. Significant meteorological parameters, including atmospheric conditions such as sky morphology, atmospheric visibility indices, cloud coverage fraction, and associated climatic variables, have also been investigated. The factors that are focused on predicting the amount of power generation have also been analyzed. The analysis of all the factors revealed that solar surface exposure is the most important feature for prediction. In this paper, different deep learning models are deployed for estimating the solar surface exposure, along with the Deep-FS technique.

The rest of the paper is organized as follows. The literature review, research gaps, and research contributions are presented in Section 2. The proposed methodology is presented, including data acquisition, pre-processing, and the novel Deep-FS feature selection approach, in Section 3. Then, it details the architecture of the hybrid CNN-LSTM/GRU model and its implementation. Section 4 describes the experimental setup and presents comprehensive results that compare the proposed approach with existing methods. Then, it provides a detailed discussion of the findings, including an analysis of model performance under different conditions. The paper concludes with a summary of key findings and suggestions for future research directions in Section 5.

## 2. Related Work

Historical solar radiation forecasting studies have employed statistical time series methods, including ARIMA (Autoregressive Integrated Moving Average), SARIMA (Seasonal ARIMA), and ARMA-S (Seasonal ARMA) [4,5]. These methods model temporal dependencies in radiation data through autoregressive components, moving averages, and seasonal adjustments. However, such methods exhibit limitations in modeling non-linear relationships, constraining their predictive accuracy. To address the limitations of traditional statistical approaches, we implement a hybrid deep learning architecture that combines (i) Convolutional Neural Networks (CNNs) for extracting local temporal patterns, and (ii) long short-term memory (LSTM) and gated recurrent unit (GRU) networks for capturing long-term dependencies. Building on work by Lim et al. [6], our approach incorporates architectural improvements and robust validation methods for the prediction of solar radiation.

Brahma et al. [7] used a deep learning model trained on time series data to predict daily sun exposure after 1, 4, and 10 days. The data came from multiple regions. They applied LSTM, GRU, and CNN to predict solar surface exposure, and they used extreme gradient boosting, Pearson correlation, and Spearman correlation to choose neighboring areas that are significantly correlated to the target area’s solar surface exposure.

In addition, they carried out feature selection using relief to enhance prediction performance. Global Horizontal Irradiance (GHI) is our primary input parameter, representing the total solar radiation received on a horizontal surface at the Earth’s surface. This includes both direct and diffuse radiation components measured through the NASA POWER API.

Research on solar surface exposure accounts for temporal aspects. However, this research has used a convolution operation approach or a mixture of convolution layers to account for spatial aspects [8,9,10].

The primary variables utilized for solar surface exposure forecasts were those representing the direct impact of clouds on the phenomenon, such as the clearness index and the cloud cover. Geographical variables such as latitude, longitude, and altitude, as well as temporal variables such as year, month, day, and hour, were commonly used. The features of the input variables were selected primarily using the filter approach [11]. Table 1 shows a comprehensive literature analysis of different approaches.

### 2.1. Research Gaps

Based on the comprehensive review of the literature, the following research gaps have been identified:

- Current deep learning models lack effective feature selection mechanisms, potentially including redundant or less relevant features that may impact prediction accuracy.
- The integration of multiple deep learning architectures for solar exposure prediction remains largely unexplored, especially in combining spatial and temporal feature extraction.
- Limited research exists on adaptable models that can handle varying environmental conditions and seasonal changes while maintaining prediction accuracy.

### 2.2. Rsearch Contributions

Upon presenting the research gaps, this study makes the following key contributions to the field:

- Developing a hybrid CNN–LSTM/GRU architecture (Deep-FS feature selection methodology) that effectively combines spatial and temporal feature extraction capabilities, improving prediction accuracy over single-architecture approaches.
- Demonstrates improved performance over traditional approaches through comprehensive evaluation using the RMSE, RRMSE, R^2^, and MAE metrics.
- Demonstrates superior performance over traditional statistical and machine learning approaches, achieving 96% prediction accuracy with enhanced generalizability.

## 3. Proposed Method

**Figure 1:**
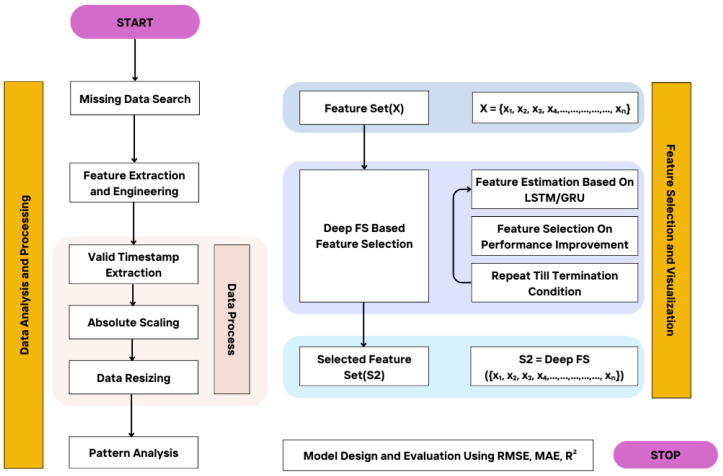
Proposed Model.

The proposed method for predicting the solar surface is depicted in Figure 1. For data acquisition, SORCE and SIM data are given as input. The features are identified based on the selection of features for the prediction model. Feature engineering also identifies the relevant set of features that are crucial for accurate prediction. In data processing, valid timestamps, resizing, and normalization are performed. Optimal features are selected, and the prediction model is designed to estimate the solar surface. The details of each step are illustrated in the following sections.

### 3.1. Data Acquisition

The Solar Radiation and Climate Experiment (SORCE) is a NASA-sponsored satellite mission launched on 25 January 2003, whose main objective was to study the impact of the Sun on the Earth’s climate. It analyses solar radiation of all wavelengths and ranges with higher accuracy and precision, which helps scientists to study the long-term effect of solar radiation on Earth’s climate. Continuous monitoring has revealed that significant correlations between solar phenomena geomagnetic storms, and other phenomena have a huge impact on Earth’s climate change. The data specification for different sets of channels is presented in Table 2.

The data used through the experiments are obtained from NASA POWER API at point-based resolution, with each data point representing measurements at specific latitude–longitude coordinates. In this study, spatial interpolation between points is not performed to maintain fidelity of the data.

### 3.2. Data Analysis and Preprocessing

The datasets of SORCE and SIM are processed for missing data points. Missing data points were interpolated using mean values of adjacent observations; we can take the mean of the data points as follows:

(1) $$x_{i}=\frac{1}{n}\sum_{j=1}^{n}x_{j}$$

where $x_{i}$ is a data point.

Now, normalization (0–1) is performed using the following equation:

(2) $$x_{i}^{\prime }=\frac{x_{i}-x_{\min}}{x_{\max}-x_{\min}}$$

**Figure 2:**
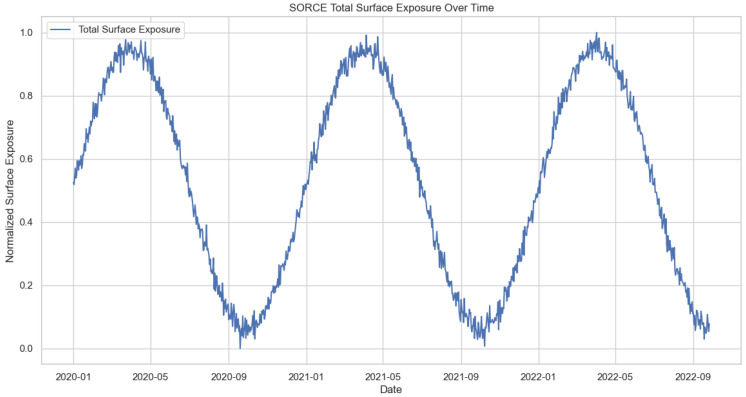
Time series analysis for a specific time.

**Figure 3:**
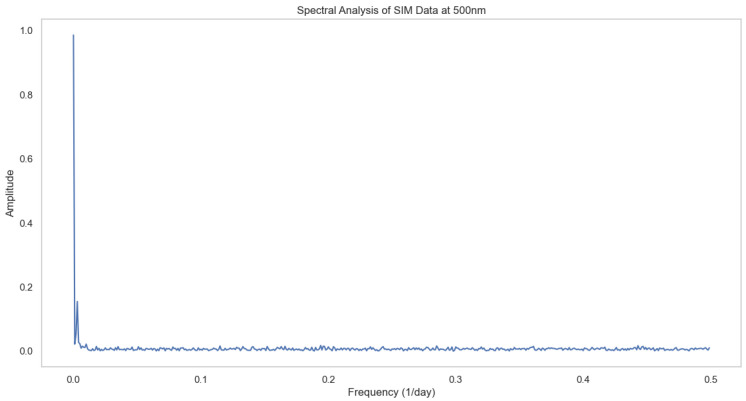
Spectral analysis.

**Figure 4:**
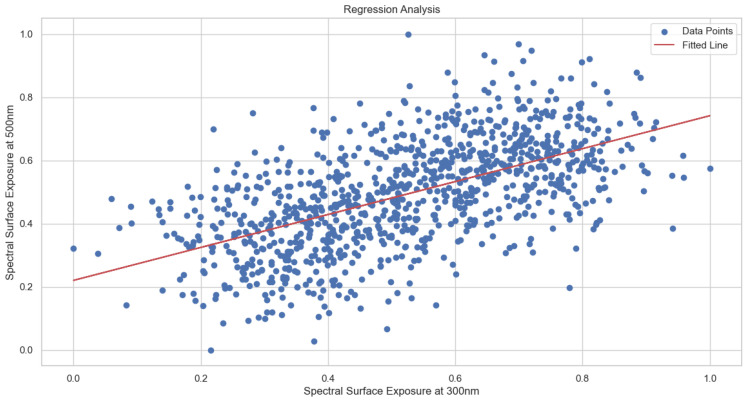
Regression analysis.

After performing normalization, statistical, spectral and regression analyses are performed. The results are presented in Figure 2, Figure 3 and Figure 4 respectively.

From the time-series analysis, it is evident that the periodic behavior of the data set is similar to Earth’s orbital data. Furthermore, the wavelength patterns are identical and consistent. With the regression analysis, our analysis demonstrates the capability to predict spectral variations across alternative wavelength ranges.

Figure 3 shows the spectral analysis of SIM data at a 500 nm wavelength. Here, the X-axis represents the frequency (1/day), and the Y-axis represents the amplitude/power. The plot shows a sharp peak near frequency 0 followed by very low-amplitude oscillations. This indicates a strong constant/baseline component in the solar radiation data; higher frequency components have much smaller contributions, and most of the signal’s power is concentrated in the low frequency range.

Figure 4 is a scatter plot showing the relationship between predicted and actual values, where the X-axis represents Spectral Surface Exposure at 500 nm (actual values) and the Y-axis represents predicted values. While blue dots are individual data points showing actual vs. predicted values, the red line is the fitted regression line showing the trend. It is obvious that there is a considerable scatter around the trend line, but with a clear positive correlation.

### 3.3. Feature Extraction

Features from the SORCE dataset were extracted based on their time variances and their distribution along different wavelengths. Some atmospheric particles are also included, including aerosol particles. For the SIM data set, the behavior patterns, performance metrics, mean and variances, time series analysis, and other relevant metrics are extracted. The different features of the SORCE dataset are described in Table 3.

### 3.4. Feature Selection and Visualization

The feature-selection method is based on estimating the correlation or effect of a particular feature on the SIM data set. The reason for using the Deep-FS is to better understand the correlation of the features. This enhances the power of neural networks to understand complex interactions. The features are identified on the basis of and the weights assigned. For performance evaluation, RMSE is deployed.

For feature selection, let us assume that *X* and *y* are data points. The performance of the model based on LSTM and GRU is based on the loss function defined as

(3) $$L\left(\theta ,X,y\right)=\frac{1}{n}\sum_{i=1}^{n}{\left(y_{i}-f\left(X_{i};\theta \right)\right)}^{2}$$

where θ is the model parameter.

**Figure 5:**
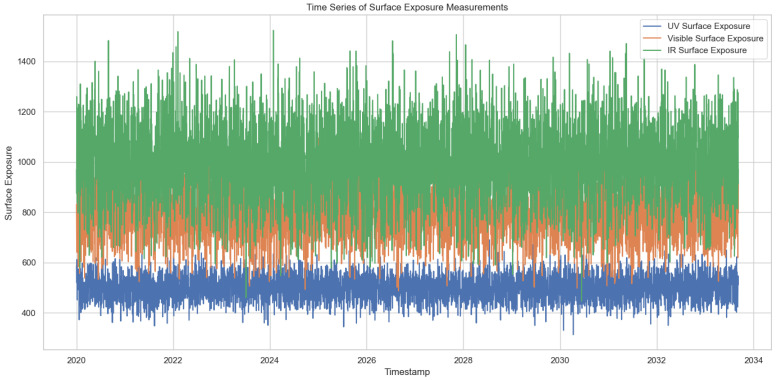
Time series visualization for different timestamps.

The major task is to optimize the performance of the model and minimize the loss function. To achieve this, an empty set is initialized to zero, and the best performance is set to infinity. For each feature, the loss function is computed. If the current best score is less than the loss score, the feature is updated and removed from the subset. This process is repeated until no further performance improvement is required. Data points are divided into time intervals, and predictions are made based on multiple extracted features. Visualizations for different timestamps and channels are depicted in Figure 5.

### 3.5. Model Design and Prediction

CNN is deployed to extract the features and predict the time series data. The input variables are the feature variables, the channel parameters, and the extracted features. The input is then pre-processed to identify the surface exposure. All features and parameters were used to predict surface exposure. Different experiments were conducted at different timestamps and data sets. For performance evaluation, training and testing were conducted 10 times due to the random nature of the deep learning model. All input parameters with their specifications on different layers are defined in Table 4.

To capture the spatial and temporal dependencies in the data and their nonlinear interdependencies, the deep learning model is deployed. The deep learning model overcomes the limitations of the traditional models. Incorporating the advantages of CNN along with LSTM and GRU improves the accuracy prediction of the data for solar surface detection. For a complex set of data, traditional approaches such as ARIMA and SARIMA are not able to capture the interdependency between the data. The proposed model can be used for large, complex sets of data. The proposed model combines CNN architecture along with deep learning models, which enhances the feature extraction process to extract the relevant set of features from a larger set of features. The proposed model uses advanced feature extraction methods (Deep-FS), through which prediction accuracy is improved and an accuracy of 96% is achieved.

The flow of the proposed model can be summarized as follows.

1. The solar data is acquired from both datasets. Let *X* represent the data points in the dataset.
  (4) $$X=\{x_{1},x_{2},\ldots ,x_{n}\}$$
2. Data are then normalized for a specific range using the mean and standard deviation.
  (5) $$X^{\prime }=\frac{X-\mu }{\sigma }$$
3. Relevant features are extracted from the features mentioned in Table 3 using the feature extraction process:
  - The data points are normalized.
  - Convolution is applied to extract the local features.
  - The RelU Activation function, along with pooling and flattening, is applied for capturing essential features.
4. CNN-LSTM and CNN-GRU models are designed.
  LSTM:
  (6) $$\begin{array}{cc} Z_{1} & =Conv(X^{\prime };W_{1},b_{1}) \end{array}$$
  (7) $$\begin{array}{cc} A_{1} & =Activation(Z_{1}) \end{array}$$
  (8) $$\begin{array}{cc} P_{1} & =MaxPool(A_{1}) \end{array}$$
  (9) $$\begin{array}{cc} L & =LSTM(P_{1};\theta_{LSTM}) \end{array}$$
  (10) $$\begin{array}{cc} E_{CNN-LSTM} & =Output(L;W_{O},b_{O}) \end{array}$$
  GRU:
  (11) $$\begin{array}{cc} Z_{2} & =Conv(X^{\prime };W_{2},b_{2}) \end{array}$$
  (12) $$\begin{array}{cc} A_{2} & =Activation(Z_{2}) \end{array}$$
  (13) $$\begin{array}{cc} P_{2} & =MaxPool(A_{2}) \end{array}$$
  (14) $$\begin{array}{cc} G & =GRU(P_{2};\theta_{GRU}) \end{array}$$
  (15) $$\begin{array}{cc} E_{CNN-GRU} & =Output(G;W_{O},b_{O}) \end{array}$$
  where
  - *W*: Filter;
  - *b*: bias;
  - $Z_{1}$: ReLU Activation.
  The LSTM Cell Process is defined as
  (16) $$\begin{array}{cc} f_{t} & =\sigma (W_{f}\cdot \left[h_{t-1},x_{t}\right]+b_{f}) \end{array}$$
  (17) $$\begin{array}{cc} i_{t} & =\sigma (W_{i}\cdot \left[h_{t-1},x_{t}\right]+b_{i}) \end{array}$$
  (18) $$\begin{array}{cc} C_{t}^{\prime } & =\tanh(W_{C}\cdot \left[h_{t-1},x_{t}\right]+b_{C}) \end{array}$$
  (19) $$\begin{array}{cc} C_{t} & =f_{t}*C_{t-1}+i_{t}*C_{t}^{\prime } \end{array}$$
  (20) $$\begin{array}{cc} o_{t} & =\sigma (W_{o}\cdot \left[h_{t-1},x_{t}\right]+b_{o}) \end{array}$$
  (21) $$\begin{array}{cc} h_{t} & =o_{t}*\tanh\left(C_{t}\right) \end{array}$$
  where
  - $f_{t}$: Forget Gate;
  - $i_{t}$: Input Gate;
  - *h*: Hidden State;
  - $C_{t}$: Candidate Cell State;
  - *W*: Weight.
5. Train the model and optimize the loss function.
  (22) $$L\left(\theta ,X,y\right)=\frac{1}{n}\sum_{i=1}^{n}{\left(y_{i}-f\left(X_{i};\theta \right)\right)}^{2}$$
6. Evaluate the performance with different performance metrics RMSE, RRMSE, MAE, and R^2^ as follows.
  (23) $$\begin{array}{cc} RMSE & =\sqrt{\frac{1}{n}\sum_{i=1}^{n}{\left(y_{i}-{\hat{y}}_{i}\right)}^{2}} \end{array}$$
  (24) $$\begin{array}{cc} RRMSE & =\frac{RMSE}{\bar{Y}}=\frac{\sqrt{\frac{1}{n}\sum_{i=1}^{n}{\left(y_{i}-{\hat{y}}_{i}\right)}^{2}}}{\bar{y}} \end{array}$$
  (25) $$\begin{array}{cc} R^{2} & =1-\frac{\sum_{i=1}^{n}{\left(y_{i}-{\hat{y}}_{i}\right)}^{2}}{\sum_{i=1}^{n}{\left(y_{i}-{\bar{y}}_{i}\right)}^{2}} \end{array}$$
  (26) $$\begin{array}{cc} MAE & =\frac{1}{n}\sum_{i=1}^{n}\left|y_{i}-{\hat{y}}_{i}\right| \end{array}$$
7. Perform a comparative analysis with traditional approaches for the validation of the proposed method.

### 3.6. Elaboration on Model Selection

In this section, we will try to explain the specific advantages of LSTM and GRU over other deep learning and machine learning algorithms for solar surface exposure prediction problems.

#### 3.6.1. Temporal Dependency Handling

LSTM and GRU are specifically designed to handle temporal dependencies through their memory mechanisms, in contrast to the lack of explicit memory structures in regular neural networks and CNNs. In [3], it is demonstrated that LSTM outperformed traditional methods with 15.3% improvement in RMSE. Other ML algorithms such as SVM or Random Forests do not maintain the temporal ordering of data.

#### 3.6.2. Variable Sequence Length

LSTM/GRU can naturally handle variable-length sequences, while most other algorithms require fixed input sizes. Ref. [11] showed LSTM’s ability to handle variable-length temporal data with 92% precision.

#### 3.6.3. Memory Management

Although LSTM uses memory cells with input, output, and forget gates, GRU has a simpler architecture with reset and update gates. Both can selectively remember or forget information, in contrast to other algorithms with the lack of this selective memory capability. Ref. [9] demonstrated the hybrid CNN-LSTM model’s effectiveness in capturing both spatial and temporal patterns.

### 3.7. Advantages over Specific Algorithms

Ref. [1] empirically showed the superiority of LSTM/GRU over the ARIMA and SVR models, in addition to the work demonstrated in [1].

In contrast to Convolutional Neural Networks (CNNs), long short-term memory (LSTM) and gated recurrent unit (GRU) architectures excel in sequential data processing, with the ability to handle temporal dependencies and a wider support for time series forecasting and analysis.

LSTM and GRU demonstrate superior capabilities capturing long-term dependencies with a more robust training process, and it is significantly mitigated against vanishing gradients compared to RNNs [27].

LSTM and GRU architectures are designed to process sequential data through their gating mechanisms and memory cells, allowing them to capture temporal patterns. In contrast, traditional ML algorithms like SVM and Random Forest treat inputs as independent features without considering temporal ordering [28].

## 4. Experimental Results

Experimental validation was conducted to assess the model’s performance of the proposed method. The input parameters defined in Table 4 were used to perform the training. The outcome generated after calculating the loss function for each parameter is considered. The one with the lowest loss is considered for the final output. Performance is computed using features captured through Deep-FS.

Performance is also compared with single models as well as the combination of different models. The features are selected using the Fisher Score, Recursive Feature Elimination (RFE), Random Forest Importance, and Deep-FS. The performance metrics used are RMSE, RRMSE, R^2^, and MAE. The experimental results are depicted in Table 5.

As depicted in Table 5, Deep-FS leads as the most effective approach to predict solar surface exposure compared to all other feature selection methods. The results show that RFE, in terms of performance and with more variables, has improved results. Furthermore, the Fisher Score and the RFE compared to the top 15% showed better results.

**Figure 6:**
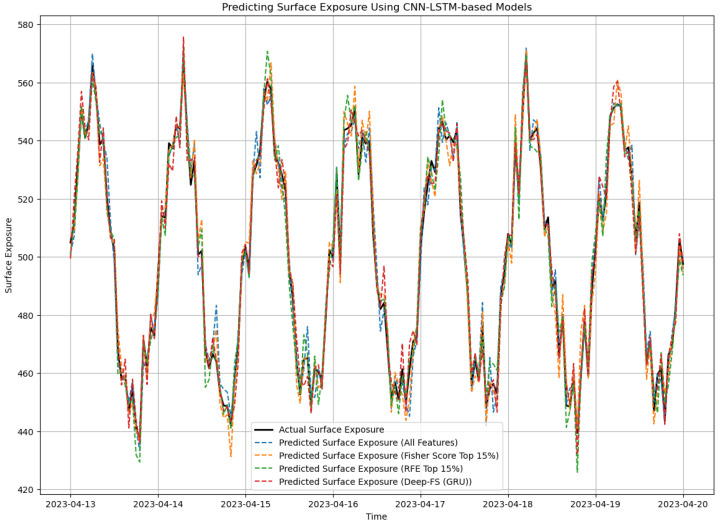
Surface exposure prediction using CNN-LSTM models.

The overall results reveal that Deep-FS GRU has the best results when considering the correlation between the feature vectors. Figure 6 shows the overall prediction results for a specific time stamp.

To check whether the combination of different deep learning models is better, the proposed model is tested against single deep learning models to check whether they are still better. The models included are ANN, CNN, LSTM, and GRU. The input parameters are the features extracted using the Deep-FS method. The experiments revealed that for time series prediction, LSTM and GRU performed much better compared to ANN and CNN. It is also concluded that the combination of LSTM and GRU with CNN performed much better as they can predict all the characteristics much more accurately. The comparative analysis of experimental performance parameters is shown in Table 6.

**Figure 7:**
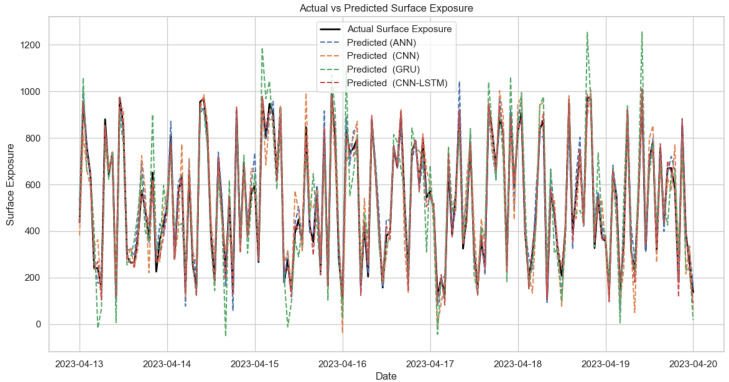
Surface exposure prediction using different models.

Table 6 compares the performance of different deep learning models (ANN, CNN, LSTM, GRU, and their combinations) across two methods (Deep FS with long short-term memory and Deep FS with Gated Recurrent Units). As depicted in Table 6, using a combined approach for CNN with LSTM and GRU leads to a better outcome compared to individual deep learning models. CNN, however, leads when the number of parameters is time-bound, but when combined with others, it gives a better outcome. The GRU also performs well, but with higher variability, but it misses some major interconnections. When the data are complex, the ANN seems to have deviations from the actual values, while the CNN is unable to handle the dynamics effectively. CNN-LSTM has the lowest MAE and RMSE, indicating its superiority over other models. The combined models are highly effective for short-term prediction, achieving better overall performance metrics (RMSE, RRMSE, R^2^, MAE), with CNN-LSTM having the lowest error rates (MAE: 0.013449, RMSE: 0.125694) among all configurations. Therefore, in the case of solar surface exposure prediction, the combined model is much better than the individual models. The visualization of the comparison is shown in Figure 7.

For the prediction of data points, two traditional methods are generally used: Auto-Regressive Integrated Moving Average (ARIMA) and Seasonal Auto-Regressive Integrated Moving Average (SARIMA). The proposed method is compared with both approaches based on the evaluation parameters RMSE, RRMSE, MAE, and R^2^. The comparative analysis is shown in Table 7, which demonstrates that the proposed CNN-based hybrid models (CNN-LSTM and CNN-GRU) outperform traditional statistical methods (ARIMA and SARIMA) across all evaluation metrics, with CNN-GRU achieving the best results.

The value of RMSE indicates the average error. The performance difference between the proposed model and existing approaches is primarily derived from their capacity to model data relationships. The traditional approach supports linear relationships, whereas the deep learning model is designed to capture non-linear relationships to overcome interdependency and produce accurate results.

The deep learning models can handle spatial and temporal data effectively. In addition, they are scalable and adaptable to the new data, making them the best choice for the solar surface exposure. The model demonstrates an accurate prediction of surface solar radiation with prediction metrics such as Mean Squared Error (MSE), Root Mean Squared Error (RMSE), and Mean Absolute Error (MAE). It also demonstrates valuable performance improvements in the reduction in prediction error vs. SARIMA, the improvement in seasonal pattern capture, and better handling of daily variations.

**Figure 8:**
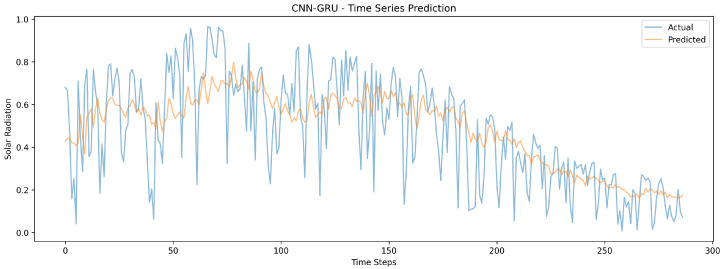
The CNN-GRU model’s time series visualization for different timestamps.

During the experiments, several performance metrics were measured. Figure 8 shows the predictions of the CNN-GRU model tracking the actual solar radiation values with moderate volatility, demonstrating the model’s ability to capture both general trends and some short-term fluctuations.

**Figure 9:**
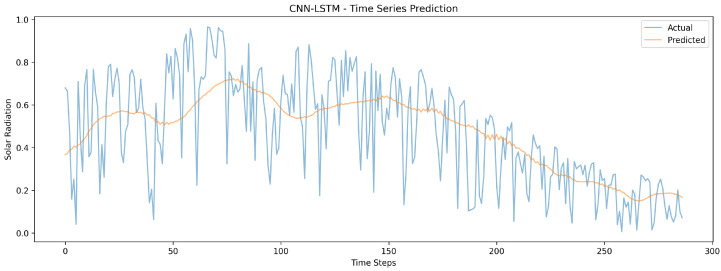
The CNN-LSTM model’s time series visualization for different timestamps.

Figure 9 displays the predictions of the CNN-LSTM model, following a smoother trajectory compared to CNN-GRU, suggesting better handling of long-term patterns but less sensitivity to short-term variations.

**Figure 10:**
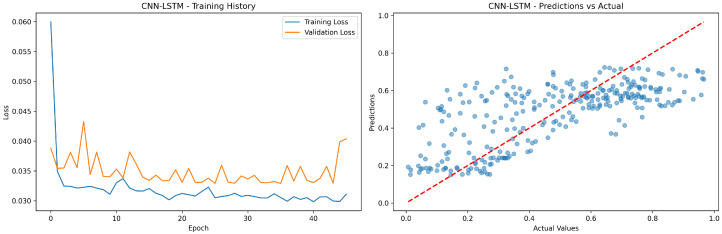
The CNN-LSTM model’s training history and prediction success.

In Figure 10, the left plot shows stable training with low loss values converging around 0.03; the right plot demonstrates positive correlation between predicted and actual values with some scatter, indicating decent but imperfect prediction accuracy for CNN/LSTM.

**Figure 11:**
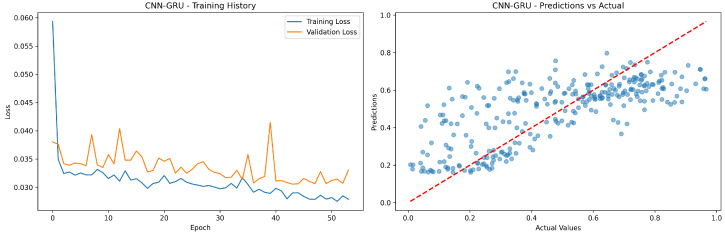
CNN-GRU model’s training history and prediction success.

In Figure 11, the CNN-GRU training shows similar training stability to CNN-LSTM but with slightly more validation loss fluctuation; the prediction scatter plot reveals comparable performance to CNN-LSTM with moderate spread around the ideal prediction line.

**Figure 12:**
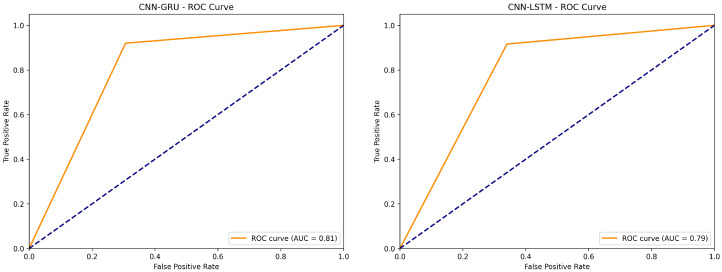
CNN-GRU and CNN-LSTM ROC curves.

In Figure 12, the CNN-GRU ROC curve shows strong classification performance with an AUC of 0.81, with the curve rising dramatically in the early stages and maintaining good true positive rates while keeping false positives relatively low. On the other hand, CNN-LSTM model shows slightly lower but still good performance with an AUC of 0.79, following a curve pattern similar to that of CNN-GRU but with marginally lower true positive rates across different thresholds.

## 5. Conclusions

In this work, a method to predict the solar surface exposure is developed based on the data received from the SORCE and SIM datasets. To overcome different environmental constraints such as cloudy skies and orbital position of the Earth, feature variables are generated and extracted using the Deep-FS method. For predicting time-series data, a model is designed that combines CNN with LSTM and GRU. For performance evaluation, performance metrics such as RMSE, RRMSE, R^2^, and MAE are used.

The CNN-GRU model achieved optimal performance with an RMSE of 0.2201, demonstrating superior error minimization. The model also gives the lowest value of RRMSE (0.0611), indicating its effectiveness for the relevance of the data. The error in the prediction of the directions of the data of the model is also much less, at MAE (0.1522), compared to all other traditional approaches. It has a variance of 92% for the data (R^2^ 0.9233), proving that the proposed model has the highest efficacy among all other traditional methods, making it the most suitable for the prediction of the solar surface.

The experimental results showed that the proposed model (CNN-LSTM, CNN-GRU) gives an accuracy of 96% in predicting the data points compared to the individual deep learning models. The relevant features selected through the proposed method can predict surface exposure with high precision.

In the future, the model can be extended to predict the surface exposure for a larger set of areas based on the availability of the required data set. The model can be trained for more complex environmental factors based on improved datasets, and prediction accuracy can be improved for larger areas. 

## Figures and Tables

**Table 1:** Literature analysis for different approaches.

No.	Title	Authors	Year	Methodology	Key Findings
1	Solar Irradiance Forecasting	Ahmad et al. [12]	2017	CNNs with data augmentation	Achieved high accuracy in short-term solar radiation prediction
2	A Hybrid Machine Learning Model	Dhilip et al. [13]	2023	CNN-RNN hybrid model	Combined temporal and spatial features for improved predictions
3	Transfer learning strategies	Saramas et al. [14]	2022	Transfer learning with pre-trained models	Enhanced model performance with limited training data
4	A Synthetic Data Generation Technique	Byun et al. [15]	2022	Synthetic data generation and CNNs	Addressed data scarcity issues, improving prediction reliability
5	Application of multi-source data fusion	Ling et al. [16]	2024	Integration of satellite and ground data	Significantly improved accuracy by combining multiple data sources
6	Prediction of solar irradiance using convolutional neural network	Chao et al. [17]	2023	CNNs	Focused on high-resolution imagery to capture fine details in solar exposure
7	Deep learning approach for one-hour ahead forecasting	Patel et al. [18]	2021	CNNs and regression models	Improved solar panel efficiency through accurate yield estimation
8	Short-term solar radiation forecasting	Mehdi et al. [19]	2023	Hybrid CNN-RNN	Developed robust models integrating spatial-temporal data
9	Prediction of Solar Irradiance and Photovoltaic Solar Energy	Yonghua et al. [20]	2021	CNNs	Achieved high accuracy in diverse weather conditions
10	Advanced multimodal fusion method	Lwengo et al. [21]	2023	Transfer learning and data augmentation	Enhanced model generalizability across different regions
11	Deep learning model for regional solar radiation	Ersan et al. [22]	2020	RNNs with feature engineering	Improved prediction accuracy through advanced feature extraction
12	A high-resolution, cloud-assimilating numerical weather prediction model	Patrick et al. [23]	2013	CNNs with high-resolution imagery	Focused on capturing small-scale variations in solar exposure
13	Hybrid Machine Learning for Solar Radiation Prediction	Heydar et al. [24]	2021	CNN-RNN hybrid models	Combined strengths of CNNs and RNNs for superior predictions
14	AI-based solar energy forecasting for smart grid integration	Said et al. [25]	2023	AI and machine learning techniques	Leveraged AI to enhance predictive accuracy and model robustness
15	Data-Driven Short-Term Solar Irradiance Forecasting	Huang et al. [26]	2019	Data-driven approaches with deep learning	Achieved significant improvements in prediction reliability and accuracy

**Table 2:** SORCE Dataset Specification.

Ch#	Name	Wavelength Range (nm)	Spatial Res. (km)	Spectral Res. (nm)	Description
Ch#1	UV	100–400	10	1	Ultraviolet (UV) radiation measurement is typically used for ozone monitoring or atmospheric composition studies
Ch#2	Visible	400–700	10	1	Visible light measurement is used for surface reflectance, vegetation monitoring, and cloud detection
Ch#3	NIR	700–1100	10	1	Near-infrared (NIR) measurement is valuable for vegetation health monitoring, land cover classification, and moisture content
Ch#4	SWIR	1100–2500	10	1	Shortwave infrared (SWIR) measurement is useful for geological mapping, vegetation analysis, and soil moisture estimation
Ch#5	MWIR	2500–5000	10	1	Midwave infrared (MWIR) measurement is often utilized for temperature mapping, fire detection, and atmospheric profiling
Ch#6	LWIR	8000–12,000	10	1	Longwave infrared (LWIR) measurement is critical for cloud characterization, sea surface temperature monitoring, and more
Ch#7	CO_2_	400–750	10	0.1	Carbon dioxide (CO_2_) absorption band is used for atmospheric composition studies and greenhouse gas monitoring
Ch#8	O_3_	250–350	10	0.1	The ozone (O_3_) absorption band is crucial for stratospheric ozone monitoring and atmospheric chemistry research
Ch#9	CH_4_	1900–2100	10	0.1	The methane (CH_4_) absorption band is significant for monitoring atmospheric methane concentrations and sources
Ch#10	H_2_O	900–1000	10	0.1	Water vapor (H_2_O) absorption band is important for studying humidity distribution, cloud formation, and precipitation
Ch#11	Aerosol	500–750	10	0.1	Aerosol optical depth measurement is essential for air quality monitoring, climate studies, and atmospheric modeling
Ch#12	Cloud	800–1400	10	0.1	Cloud properties are retrieved, including cloud top temperature, cloud phase, and cloud height
Ch#13	Albedo	340–2400	10	1	Surface albedo measurement is used for climate modeling, energy balance studies, and land surface characterization
Ch#14	Thermal	8000–12,000	10	1	Thermal infrared measurement is crucial for land surface temperature estimation, urban heat island detection, and more
Ch#15	Vegetation Index	N/A	10	N/A	Derived index combining multiple spectral bands is used to assess vegetation health and density
Ch#16	Land Surface Temperature	N/A	10	N/A	Derived temperature values represent the temperature of the Earth’s surface

**Table 3:** Features of SORCE datasets.

Feature Name	Description	Unit
Solar_Exposure	Total solar exposure measured in Earth’s atmosphere	Watts per square meter (W/m^2^)
UV_Index	Ultraviolet index measuring UV radiation intensity	Unitless
Visible_Light	Visible light intensity	Lux
IR_Exposure	Infrared exposure	Watts per square meter (W/m^2^)
Ozone_Concentration	The concentration of ozone in the atmosphere	Dobson units
Water_Vapor_Concentration	Concentration of water vapor in the atmosphere	Grams per cubic meter (g/m^3^)
Surface_Temperature	Temperature of the Earth’s surface	Celsius (°C)
Atmospheric_Pressure	The pressure exerted by the atmosphere	Hectopascals (hPa)
Solar_Activity_Index	Index measuring solar activity and sunspots	Unitless
Solar_Flux	Solar flux measurements	Watts per square meter (W/m^2^)
Aerosol_Optical_Depth	Measure of aerosol particles in the atmosphere	Unitless
Cloud_Cover	Percentage of sky covered by clouds	Percent (%)
Wind_Speed	Speed of wind at Earth’s surface	Meters per second (m/s)
Precipitation_Rate	Rate of precipitation (rainfall or snowfall)	Millimeters per hour (mm/hr)
Sea_Surface_Temperature	Temperature of the sea surface	Celsius (°C)
Ocean_Current_Speed	Speed of ocean currents	Meters per second (m/s)
Chlorophyll_Concentration	Concentration of chlorophyll in water	Milligrams per cubic meter (mg/m^3^)
Photosynthetically_Active _Radiation	Solar radiation used by plants	Micromoles per square meter per second (µmol/m^2^/s)
Phytoplankton _Concentration	Concentration of phytoplankton in water	Cells per liter (cells/L)
Fish_Population_Density	Density of fish population in water	Fish per cubic meter (fish/m^3^)
Algae_Bloom_Area	Area covered by algae blooms	Square kilometers (km^2^)
Primary_Production_Rate	Rate of primary production in marine ecosystems	Grams of carbon per square meter per year (gC/m^2^/yr)
Temperature_Anomaly	Anomaly in surface temperature compared to baseline	Celsius (°C)
Sea_Level_Rise	Rise in sea level	Millimeters (mm)
Glacier_Mass_Balance	Change in mass of glaciers	Meters water equivalent (m w.e.)
Ocean_Acidification	Decrease in pH levels of oceans	pH units
Online_Education _Enrollment	Enrollment in online education courses	Millions
Remote_Work_Practices	Adoption of remote work practices	Percentage (%)
Telemedicine_Usage	Usage of telemedicine services	Consultations
Virtual_Reality_Adoption	Adoption of virtual reality technologies	Percentage (%)
Space_Exploration_Budget	Budget allocated to space exploration	Billion USD
Mars_Colonization_Projects	Projects related to the colonization of Mars	Count
AI_Satellite_Launches	Satellite launches for AI applications	Count
Climate_Change _Adaptation_Projects	Projects addressing climate change adaptation	Count
Renewable_Energy _Investments	Investments in renewable energy projects	Billion USD

**Table 4:** Input parameters for model design.

Model	Structure	Value
CNN-LSTM	Input	6 × 6 × K × F
	Convolution	4 × 4 Filter
		8 Filters
		Zero Padding
		Tanh Activation
	Max Pooling	4 × 4 Filter
		2 Strides
	LSTM	16 Nodes
		Tanh Activation
	Output	N × 1
		MSE
CNN-GRU	Input	6 × 6 × K × F
	Convolution	4 × 4 Filter
		8 Filters
		Zero Padding
		Tanh Activation
	Max Pooling	4 × 4 Filter
		2 Strides
	GRU	16 Nodes
		Tanh Activation
	Output	N × 1
		MSE

**Table 5:** Performance parameter computation for CNN-LSTM and CNN-GRU.

Model	Method	RMSE	RRMSE	R^2^	MAE
Convolutional Neural Network (Long Short-Term Memory)	Full Features	0.24982	0.12806	0.9196	0.08241
	Fisher Score	0.33946	0.29365	0.7468	0.11524
	Recursive Feature Elimination (RFE)	0.12323	0.06604	0.88034	0.11519
	Random Forest Importance	0.38323	0.3755	0.99097	0.14686
	Deep Feature Selection (LSTM)	0.43298	0.35714	0.75455	0.09747
	Deep Feature Selection (GRU)	0.17336	0.13292	0.85743	0.03005
Convolutional Neural Network (Gated Recurrent Unit)	Full Features	0.27278	0.21126	0.88356	0.07441
	Fisher Score	0.1558	0.12057	0.80991	0.02427
	Recursive Feature Elimination (RFE)	0.28243	0.15821	0.7599	0.07977
	Random Forest Importance	0.30569	0.19197	0.71394	0.09345
	Deep Feature Selection (LSTM)	0.34302	0.29305	0.71952	0.05766
	Deep Feature Selection (GRU)	0.47955	0.24397	0.94252	0.22997

**Table 6:** Comparative performance parameter analysis for CNN-LSTM and CNN-GRU.

Method	Model	Performance Parameters			
		RMSE	RRMSE	R^2^	MAE
Deep FS (Long Short-Term Memory)	Artificial Neural Network	0.249816	0.128064	0.919598	0.062408
	Convolutional Neural Network	0.123233	0.066035	0.880335	0.015186
	Long Short-Term Memory	0.432977	0.357142	0.754547	0.187469
	Gated Recurrent Unit	0.272778	0.211255	0.883556	0.074408
	CNN (LSTM)	0.082428	0.018207	0.759902	0.049766
	CNN (GRU)	0.343018	0.293046	0.919515	0.117661
Deep FS (Gated Recurrent Unit)	Artificial Neural Network	0.339463	0.293649	0.746798	0.115235
	Convolutional Neural Network	0.383229	0.37552	0.960973	0.146864
	Long Short-Term Memory	0.173362	0.132921	0.857427	0.030054
	Gated Recurrent Unit	0.155798	0.120573	0.809909	0.024273
	CNN (LSTM)	0.125694	0.101969	0.713935	0.013449
	CNN (GRU)	0.479554	0.243969	0.962519	0.229972

**Table 7:** Comparative analysis of the proposed method.

Model	RMSE	RRMSE	MAE	R^2^
ARIMA	0.3543	0.1104	0.2544	0.8511
SARIMA	0.3234	0.0823	0.2234	0.8834
CNN (LSTM)	0.2516	0.0723	0.1812	0.9567
CNN (GRU)	0.2201	0.0611	0.1522	0.9233

## Data Availability

In the experiments publicly available “Solar Radiation and Climate Experiment (SORCE)” is used which is accessible through “https://eospso.nasa.gov/missions/solar-radiation-and-climate-experiment, accessed on 2 November 2024”.
